# Psychometric characteristics of the FertiQoL questionnaire in a German sample of infertile individuals and couples.

**DOI:** 10.1186/s12955-018-1058-9

**Published:** 2018-12-17

**Authors:** R. E. Sexty, G. Griesinger, J. Kayser, M. Lallinger, S. Rösner, T. Strowitzki, B. Toth, T. Wischmann

**Affiliations:** 1 Institute of Medical Psychology, Centre for Psychosocial Medicine, University Hospital, Bergheimer Strasse 20, 69115 Heidelberg, Germany; 2 Department of Gynaecological Endocrinology and Reproductive Medicine, University Hospital of Schleswig-Holstein, Campus Lübeck, Lübeck, Germany; 3 Department of Gynaecological Endocrinology and Reproductive Medicine, Women’s Hospital, University Hospital, Heidelberg, Germany; 4 Department of Gynaecological Endocrinology and Reproductive Medicine, Medical University Innsbruck, Innsbruck, Austria

**Keywords:** Fertility-specific QoL, Validation, German sample, Sociodemographic variables, Cross-partner effects

## Abstract

**Background:**

FertiQoL is a questionnaire internationally developed to measure fertility-specific quality of life. It has been validated with infertile populations in many countries and used in several studies focusing on the psychosocial consequences of infertility in Europe, Asia, and North America.

**Methods:**

Over a period of two years, 596 infertile women and men took part in the study conducted at three German fertility clinics. Psychometric properties of FertiQoL were tested by performing confirmatory factor analyses, calculating average variance extracted values, reliability and correlation coefficients. Hierarchical regression analyses were conducted to determine the relations between FertiQoL subscales and both sociodemographic and medical variables. Individual and cross-partner effects were tested for.

**Results:**

The confirmatory factor analyses conducted on our FertiQoL data supported the original four-factor solution for both women and men but, resulted in some unsatisfactory indices. Family and friends’ support items loaded weakly on the Social subscale of FertiQoL (.27 and .34 in women, .32 and .19 in men). The Emotional and Mind/Body subscales revealed a strong intercorrelation (*r* = .77, *p* < .001 in women, *r* = .74, *p* < .001 in men). Women scored lower than men on the Emotional and Mind/Body subscales only, and they reported better fertility-specific relational QoL. In women, the perceived cause of infertility and already mothering a child related significantly to individual FertiQoL scores, while in men, age, educational level, and the duration of their wish for a child had an impact on the FertiQoL subscales (all *p* < .05). The men’s educational level, the women’s educational level, and the subjective perceived medical cause of fertility problems exerted cross-partner effects on QoL (all *p* < .05).

**Conclusions:**

Our study results represent a contribution both to research and clinical practice. The findings suggest the importance of considering the personal experience of infertility in different cultural and gender specific settings and that the strong connections between the emotional, physical, and cognitive aspects of an individual’s fertility-specific quality of life should be regarded as a more coherent system.

**Trial registration:**

DRKS: DRKS00014707. Registered 1 May 2018 (retrospectively registered).

## Introduction

Infertility is medically defined as the inability to conceive within a defined period (1–2 years) by persons who have regular heterosexual intercourse in the absence of contraception [1]. Depending on the criteria underlying the definition and the methodology employed in the statistical analysis, about 48.5–74.4 million women are affected by fertility problems worldwide [2, 3]. Half of these infertile individuals seek medical help for their fertility intentions [3]. There has also been an increase in the number of assisted reproductive technology (ART) cycles performed annually in Europe [4] and around the globe [1].

Decades of research [5] have exposed infertility-related emotional and cognitive responses affecting the psychosocial well-being [6], partnership dynamics [7], adjustment to periods of fertility treatment [8], and treatment- and life-related decisional behaviour [9] displayed by the individuals concerned [for an overview: [10, 11]. Psychosocial counselling as an integral component in fertility treatment is highly valued as a way of identifying patients’ emotional impairments, relational instability, discomfort in their social and cultural environment, or shortcomings in their communication with medical staff [11].

As opposed to generic psychological instruments, fertility-specific measurements give a more accurate picture of the cognitive, behavioural, and emotional state of persons with fertility problems because of the specific relationship they have to the problems and conditions involved [11, 12]. FertiQoL was developed on the basis of health psychology to measure the fertility-specific quality of life (QoL) of individuals with fertility impairments, regardless of their specific fecundity disorder, gender, or sociocultural background [13]. The Core part of the tool consists of four subscales which focus on fertility-related Emotional, Mind/Body, Relational, and Social life domains of the individuals struggling with fertility problems. The questionnaire has been devised to establish itself as a useful resource both in research and in clinical practice worldwide. The instrument has demonstrated good construct validity with satisfactory psychometric properties across a variety of national samples [14, 15] and has also displayed convincing convergent validity with depression-, anxiety- and relationship-specific scales [15–18].

Furthermore, it has been established that several sociodemographic and medical factors have specific and well-defined relations to the domains of FertiQoL. The QoL of infertile men (compared to infertile women) and both female and male study members already parenting children were more positive [13] except in the relational domain [14, 19–22]. Older persons were more likely to report better QoL [19, 23], as were people with a shorter history of infertility [21] or without any experience of fertility treatments or ART [19, 22]. In many [18, 19, 21, 22]- but not in all cases [23]- a higher educational level is associated with for better adjustment to fertility problems. Some studies have found a more positive connection between higher QoL and unexplained infertility compared to all other infertility diagnoses [19, 24], whereas infertility diagnoses with a male- or female-only factor correlated with better QoL scores than combined or unexplained factors in other investigations [18] [25]. Perceived or desired psychosocial support correlated with worse QoL [19, 23], and other medical treatment, difficulties in connection with hospital visits, unemployment, and problems in other fields of life also indicated worse QoL [19].

The aim of this cross-sectional study was twofold. First, we investigated the psychometric properties of the FertiQoL in a German sample of men and women struggling with fertility problems. The first study question was – since the FertiQoL tool had not been validated in a German sample before – whether the four-factor structure of the questionnaire [13] fitted the German study-population data. The proposal was made to measure the convergent and discriminant validity of FertiQoL in groups of women and men. Second, we investigated the connections between the different facets of fertility-related quality of life (FertiQoL subscales) and the sociodemographic and medical characteristics of individuals and their partners. Our hypothesis was that age, educational level, duration of desire for a child, parenthood, prior-to-treatment status, and (perceived) cause of infertility might be closely related to QoL of the individuals [14, 19]. A growing body of research has revealed that infertility-related concerns are influenced by the depressive symptoms, attachment style, coping style, and perceived social support from the partner [25–28]. However, interrelations between sociodemographic and medical data and psychosocial well-being have yet to be examined at the partnership level. Third, as infertility is a shared stressor within couple relationships, we set out to identify how these potentially significant factors related to fertility-specific quality of life of the two partners constituting a couple.

## Methods

### Participants and procedure

Women and men with infertility problems attending German fertility centres were sampled. Data was collected from two departments specializing in gynaecological endocrinology and fertility disorders at university-based clinics and one private fertility centre over a period of two years between December 2011 and November 2013. In consecutive sampling, potential participants were recruited by the administrative staff and asked to fill out the questionnaire package while waiting for medical consultation or examination. Individuals and couples were attending a centre for the first consultation or were about to embark on their first ART cycle at the respective institute. Inclusion criteria were fertility problems of at least twelve months, age over 18 years, and sufficient knowledge of the German language to be able to complete the questionnaire. Informed consent documents were signed by all study members after being informed about the aim of the study, how their data would be managed, and their right to quit the investigation at any time without consequences for their further medical care. Participation was voluntary, and codes were assigned to each study member to preclude identification. The Ethics Committee of the Medical Faculty of the Ruprecht-Karls University Heidelberg provided ethical approval for the study for the responsible and coordinating study centre in Heidelberg. The trial has been retrospectively registered in www.drks.de with the registration number DRKS00014707.

### Materials

The German version of International FertiQoL was assessed for the study (http://sites.cardiff.ac.uk/fertiqol/files/2015/02/fertiqol-German.pdf). Of the two modules of FertiQoL, only the Core module was involved in the study. The Core module contains 24 items organized into four subscales and a Global scale.

The Emotional subscale covers infertility-related negative emotions (e.g. “Do you experience grief and/or feelings loss about not being able to have a child?”). Mind/Body items target cognitive and physical complaints caused by reproductive problems (e.g. “Do you feel pain and physical discomfort because of your fertility problems?”). The Relational subscale refers to fertility-specific issues experienced within the couple relationship (e.g. “Do you find it difficult to talk to your partner about your feelings related to infertility?”). The Social subscale relates to perceived social support, social expectations and feelings of social isolation and shame resulting from infertility (e.g. “Do you feel uncomfortable in attending social situations like holidays or celebrations because of your fertility problems?”). The Global scale was calculated by adding the four subscales’ scores and dividing by four. Participants are asked to rate how frequent or how strong the particular statements reflect their feelings and thoughts. The Likert-type item format consists of 5 choices (0–4). Higher scores indicate better fertility-specific quality of life.

A background information form inquired into sociodemographic data (e.g. age, education, marital status, duration of relationship, duration of desire for a child), fertility-related medical data (e.g. duration of treatment, type of treatment, existence of a child from a previous or current relationship). Also, a question designed to elicit subjective fertility-related information was added to the question form: “What do you think is the cause of your infertility?” The number of potential responses was restricted to the following choices of the perceived diagnosis: “There is no cause”, “Female factor only”, “Male factor only”, “Combined factor” or “I don’t know”.

### Statistical analyses

Descriptive analyses were used to calculate means, standard deviations, and frequencies in participants’ sociodemographic and medical characteristics.

To test the original four-factor structure of FertiQoL Core in our German sample, we first conducted a confirmatory factor analysis (CFA). Goodness of fit was evaluated by chi-squared (measuring the discrepancy between the original model and our empirical data), the standardized root-mean-square residuals (SRMR, assessing averaged residuals between the original model and the observed data), root-mean-square error of approximation (RMSEA, assessing differences between the two models taking into account the error of approximation in the sample), and comparative fit index (CFI, a discrepancy function adjusted to sample size). The fit of a model is considered good if chi-squared is close to zero, ideally non-significant (*p* > 0.05), the SRMR is below 0.08, the RMSEA is below 0.06, and CFI is over 0.9/0.95 [29].

To assess the convergent validity of the four-factor model, we computed the average variance extracted (AVE) with the use of the standardized factor loadings and item numbers and the composite reliability (CR) with the use of the standardized factor loadings and error variances [30]: AVE is satisfactory >.50, CR‘s critical threshold is >.70. The reliability of the factors was also identified by computing internal consistency coefficients for each subscale divided into female and male subsamples. A Cronbach’s alpha value of >.70 was considered satisfactory for the subscales [31]. The correlation between each item and between the items and their specific factors was also analysed. To explore the discriminant validity of the instrument for the German sample, Pearson’s correlation coefficients were also calculated (a) between the subscales and (b) between the subscales and the total scale. A correlation of >.70 was assumed to represent conceptual overlap. Another method was tested to assess discriminant validity using the AVE of each factor and the shared variance of other factors. Discriminant validity was proved when the AVE of a specific factor was larger than the shared variance between the specific factor and each other factor [30].

To detect associations between the subscales of FertiQoL and sociodemographic and medical characteristics of the individuals, hierarchical regression analyses with stepwise method were conducted for men and women separately. In the first model, independent variables were individual age, educational level (low, middle, high), child from previous or current relationship, subjective cause of infertility (none, female factor only, male factor only, combined factor, unknown), prior-to-treatment status, and duration of the partnership and the desire for a child. In the next model, regression analyses testing both the individual and cross-partner effects of sociodemographic and medical aspects on QoL were performed on couples. In this second model, defined variables from both the individual and the partner were entered, including age, education level, and perceived cause of infertility. Additionally, couple variables as child from previous or current relationship, prior-to-treatment status, and duration of the partnership and the desire for a child were added as predictors as well.

Confirmatory factor analyses were captured by SAS, version 9.4 (SAS Inc., Cary, NC, USA). All other analyses were performed by IBM SPSS for Windows, version 20 (Armonk, NY: IBM Corp), with the level of significance set at *p* < 0.05.

A total of 362 women (response rate 85%) and 234 men (response rate 76%) agreed to participate in the study and returned the completed assessment sheets. Of the total number of participants, *N* = 462 (78%) study members took part as couples in the study.

## Results

In the final sample, the numbers of women and men involved differed considerably (*N* = 362 and *N* = 234 respectively). Table 1 shows the sociodemographic and medical data for the sample.

**Table 1:** Sample characteristics

	M (SD)
Age	35.58 (5.46)
Duration of partnership (yrs)	8.21 (4.77)
Duration of child wish (yrs)	3.2 (2.41)
Duration of treatment (yrs)^a^	.88 (1.29)
	*N* (%)
Gender	
Woman	362 (61)
Men	234 (39)
Education	
Low	200 (34)
Medium	119 (20)
High	277 (46)
Married	455 (76)
Own child of the woman (yes)	36 (6)
Own child with partner (yes)	51 (9)
Own child of the man (yes)	46 (8)
Prior to treatment^b^	462 (78)
Subjective cause of infertility^c^	
None	51 (9)
Female factor	117 (20)
Male factor	100 (17)
Mixed factor	143 (24)
I don’t know	170 (30)

^a^missing data was not imputed, *N* = 372

^b^abscence of any kind of fertility treatment including ovulation induction, intrauterine insemination and ART

^c^*N* = 584

The significant chi-squared indicated a major difference between expected and observed covariance matrices for both genders [women: chi^2^ (246) = 597.64, *p* < .001; men: chi^2^ (246) = 501.99, *p* < .001]. Other fit indices also were less than satisfactory too (women: RMSEA = .06, SRMR = .07, CFI = .89; men: RMSEA = .07, SRMR = .08, CFI = .88).

**Fig. 1:**
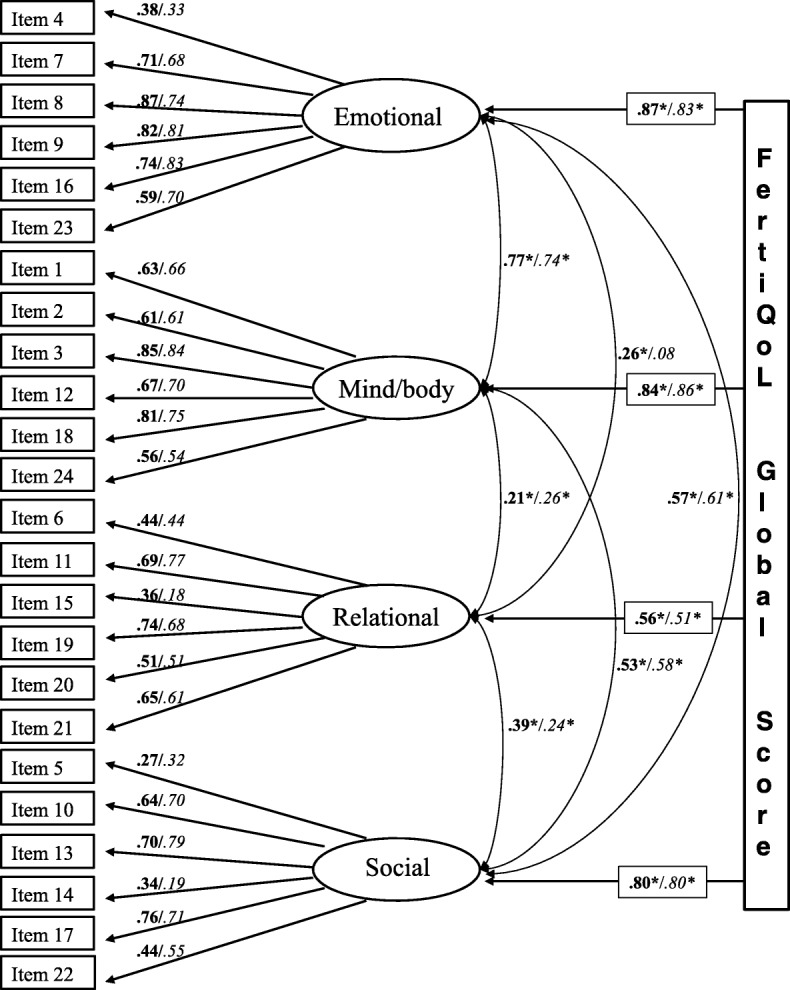
The four factor model of FertiQoL. Standardized factor loadings and correlations of subscales are presented. **Bold**: women, *italic*: men * = *p* < 0.001

All the standardized factor loadings of FertiQoL ranged between .27 and .87 in women and between .18 and .84 in men (Fig. 1). As an indicator of convergent validity, the average variances extracted explained by Emotional and Mind/Body subscales were near the critical threshold of .50 (with .50 and .49 in women and .49 and .47 in men, respectively). But, the AVE values of the Relational and Social subscales were smaller than satisfactory (with .34 and .31 in women and .31 and .36 in men, respectively). Regarding discriminant validity, Emotional and Mind/Body subscales did not show significant difference, as their AVE values were not greater than the shared variance (squared correlation estimate) of the two subscales in both genders (squared correlation estimates was .59 in women and .56 in men). The value of AVE of the Social subscale was also not greater than the shared variance of the Social and Emotional subscales (.32 in women, .37 in men), either. In all other cases, the AVE of a certain subscale was above the shared variance (ranged between .006 and .34) of the correlations of the subscales supporting the discriminant validity of each subscale. Intercorrelations among the subscales were significant but wide-ranging (Pearson’s coefficients from .21 to .77), confirming that the subscales represent separate but related dimensions of fertility-specific quality of life (Fig. 1). Only the correlation between Relational and Emotional subscales in men was not significant (Pearson’s coefficient = .08).

Because of the poor goodness-of-fit and convergent validity values, we ran the CFA again without items with standardized factor loading below .50 (items 4, 5, 6, 14, 15, 22 were excluded), and calculated the AVE values in the total sample. As expected, some fit indices were improved: SRMS = 0.05, CFI = 0.93. The AVE value of the Emotional subscale was satisfying (.57), and the AVE value of Relational and Social subscales got closer to the threshold (.42 and .49, respectively).

Table 2 presents the reliability analysis of the FertiQoL subscales: the Cronbach’s alpha values for all subscales ranged from .65 (Relational) to .84 (Emotional) for men and from .68 (Social) to .84 (Mind/Body) for women; the Composite Reliability is above .70 for Emotional, Mind/Body and Social subscales in both genders. All subscales were normally distributed (data not shown). As expected, all subscales had a moderate to strong correlation with the Global scale (Pearson’s coefficients ranged from .51 to .87).

**Table 2:** Descriptive statistics and reliability coefficients of FertiQoL subscales

	Women (*N* = 362)				Men (*N* = 234)			
	Cronbach’s alpha coefficient	Composite Reliability	Mean score (SD)	Correlation with FertiQoL Global	Cronbach’s alpha coefficient	Composite Reliability	Mean score (SD)^a^	Correlation with FertiQoL Global
Emotional	.83	.85	61.3 (18.7)	.87**	.84	.85	75.4 (16.1)***	.83**
Mind/Body	.84	.85	74.3 (14.5)	.84**	.83	.84	82.8 (14.0)***	.86**
Relational	.70	.09	79.3 (14.1)	.56**	.65	.09	76.5 (14.0)*	.51**
Social	.68	.71	75.6 (15.6)	.80**	.67	.75	76.4 (13.4)	.80**
Global	.89	.94	72.6 (12.8)	–	.87	.94	77.8 (10.8)***	–

^a^compared with mean scores of women (using independent t-tests)

***p < 0.001 **p < 0.01 *p < 0.05s

As for differences between genders, the men scored higher on the Emotional and Mind/Body subscales and the Global scale [75.4 ± 16.1 vs. 61.3 ± 18.7 t(594) = 9.446, *p* < 0.001; 82.8 ± 14.0 vs. 74.3 ± 14.5 t(594) = 6.302, *p* < 0.001; and 77.8 ± 10.8 vs. 72.6 ± 12.8 t(594) = 5.058, *p* < 0.001], whereas women did better in the relational domain [79.3 ± 14.1 vs. 76.5 ± 14.0, t(499) = 2.412, *p* < 0.05]. No gender difference was detectable in the social quality of life subscale.

Table 3 shows the individual and cross-partner effects of sociodemographic and medical characteristics on the FertiQoL subscales. In the women’s case, already being a mother was positively associated with Emotional (ß = .152, *p* < .01), and Mind/Body quality of life (ß = .132, *p* < .05), whereas already having a child with the partner was negatively associated with Relational QoL (ß = − 0.127, *p* < .05). Emotional QoL was higher when the cause of infertility was perceived as male factor only (ß = − 174, *p* < .01) or was reported as “I don’t know” (ß = .155, *p* < .01), and Mind/Body QoL was lower when the women cited combined factor (ß = −.223, *p* < .001) or female factor only (ß = −.194, *p* < .01) as the cause of fertility problems, In men, age was positively related to the emotional and Mind/Body domains of FertiQoL (ß = .258, *p* < .001; ß = .182, *p* < .01). A low level of education was found to be a negative predictor of Mind/Body and Relational QoLs (ß = −.151, *p* < .05; ß = −.15, *p* < .05). Emotional and Social QoLs were negatively associated with longer durations of wanting to have a child (ß = −.159, *p* < .05; ß = −.192, *p* < .01).

**Table 3:** Individual and cross-partner effects of sociodemographic and medical variables on FertiQoL subscales (for men and women separately)

FertiQoL subscales	Women (*N* = 362)					Men (*N* = 234)				
	Adjusted R^2^	Variable(s)	ß	t	*p*	Adjusted R^2^	Variable(s)	ß	t	*p*
Individual effects										
Emotional	.051	Own child	.152	2.803	<.01	.091	Age	.258	3.796	<.001
		Don’t know the cause	.174	3.113	<.01		Combined factor	−.159	−2.341	<.05
		Male only factor	.155	2.801	<.01					
							Duration of child wish	−.159	−2.313	<.05
Mind/Body	.056	Combined factor	−.223	−3.819	<.001	.076	Age	.182	2.700	<.01
		Female only factor	−.194	−3.318	<.01		Combined factor	−.168	−2.494	<.05
		Own child	.132	2.441	<.05		Low education	−.151	−2.229	<.05
Relational	.037	Prior to treatment (vs. in treatment) status	.142	2.572	<.05	.018	Low education	−.15	−2.165	<.05
		Own child with partner	−.127	−2.296	<.05					
Social	–	–	–	–	–	.032	Duration child wish	−.192	2.788	<.01
Individual and cross-partner effects^a^										
Emotional	.031	Don’t know the cause (F)	.158	2.252	<.05	.099	Age (M)	.265	3.905	<.001
							Combined factor (F)	−.186	−2.758	<.01
							Duration of child wish	−.15	−2.217	<.05
		Own child	.14	2.006	<.05					
Mind/Body	.034	Combined factor (F)	−.159	−2.284	<.05	.089	Age (M)	.196	2.887	<.01
		Own child of the man	−.143	−2.052	<.05		Combined factor (F)	−.203	−3.002	<.01
							Low education (M)	−.156	−2.303	<.05
Relational	.038	Low education (M)	−.207	−2.981	<.01	.044	Low education (F)	−.187	−2.685	<.01
							Male factor only (F)	.153	2.204	<.05
Social	.021	Low education (M)	−.147	−2.099	<.05	.032	Duration of child wish	−.192	−2.759	<.01

^a^individual and cross-partner mixed effects were calculated for couples only (*N* = 231 couples)

(F) female’s effect

(M) male’s effect

Underlined variables show that a variable was a predictor in both individual and individual/cross-partner settings

In the second regression model, both individual and cross-partner effects on the specific FertiQoL subscales were examined in the couples participating in the study (*N* = 231 couples). Of the cross-partner effects, only low educational level of men turned out to be a significant negative predictor for the Relational subscale (ß = −.207, *p* < .01) and the Social subscale (ß = −.147, *p* < .05) in women. In men, the combined factor perceived by the partner as the cause of infertility was a predictor for a lower level of Emotional and Mind/Body QoL in men (ß = −.186, *p* < .01; ß = −.203, *p* < .01). On the Relational subscale of FertiQoL, only cross-partner effects mattered for men, i.e. low education on the woman’s part (ß = −.187, *p* < .01) and a male infertility factor perceived by the woman (ß = .153, *p* < .05).

## Discussion

This study examines the factor structure and psychometric properties of the FertiQoL questionnaire in a sample of German women and men attending fertility clinics. Both at individual and cross-partner levels, it reveals correlations between aspects of quality of life and sociodemographic characteristics. All the analyses were conducted for men and women separately in order to compare our results with finding of previous validation studies conducted exclusively or at least primarily among women (c.f. [13, 16]).

The convergent validity of the instrument was partially satisfying as the Global scale and the Emotional and Mind/Body subscales showed very good values for Cronbach’s alpha and the Composite Reliability (above .83). The factor loadings were mostly above .70 on these two subscales. The internal consistency of the Relational subscale in men and the Social subscale in both gender groups was under .70. The reliability of the Relational subscale in men would improve to .71 if question 15 (“not strengthen relationship”) were deleted, similarly to the results of Donarelli et al., 2016 [15], and the Social subscale in men would show a Cronbach’s alpha of .74 without question 14 (“satisfaction with family support”). The Social subscale would not improve for women if any items were deleted. A similarly weak loading of satisfaction with family support as in our sample has also been captured in infertile populations in Italy and Iran [15, 18]. The CFA without items of low factor loadings resulted in better fit indices and AVE values. From the six deleted items, three were connected to Social and two to the Relational subscales. The poor convergent validity of Relational and Social subscales may root in the fact that they focus on interpersonal relations which are multifaceted and more complex phenomena than someone’s own feelings and cognitions. The problematic loading of family support in connection with fertility problems can be inferred from the fact that the greatest social support for involuntary childless persons is generally provided by the partner, not by the family [32, 33], and the amount of perceived support from significant others depends on the kind of relationship obtaining in each case, which covers a spectrum ranging from close to distant. It is worth mentioning at this point that satisfaction with friends’ support also may be hard to define for the participants in our study as it got low loadings in the social domain for both genders as well.

In relation to the discriminant validity, the subscales were identified as different constructs in most cases. Specifically the Relational subscale had only a weak correlation with all other subscales and even did not correlate significantly with the Emotional subscale in the case of men. However, the Emotional and Mind/Body subscales showed strong interrelations based on calculations with the AVE values and high correlation coefficients. The Social subscale turned out to have a strong relation to the Emotional dimension, as well. This suggests that in a German sample of infertile population, emotional and physical reactions should be strongly connected on the personal level and some emotional reactions can hardly be separated from social experiences.

On the Emotional and Mind/Body subscales and the Global scale of FertiQoL, our findings reveal gender differences that are in concordance with the findings of previous FertiQoL studies [14, 19–22]. In our investigation, no differences were found in the Social subscale. The women reported better QoL with regard to relational issues matching the trends reported by previous studies [34, 35]. The same evidence assessed with the WHO-HRQOL questionnaire in both general and infertile populations [25, 36] showed the men scoring higher on the psychological scale and women reporting better social QoL. In international comparison, the German participants scored quite high (above 70 points) on FertiQoL Global [24]. Keeping this in mind, our findings also show that German women tend to experience as high levels of fertility-related QoL as men in the relational and social domains, and do not suggest that German men report worse QoL than men with fertility problems in other countries. However, further investigations should be conducted to uncover why the expected gender differences fail to be revealed in this study.

Our hypothesis that some sociodemographic and medical variables had an impact on the quality of life of individuals and even on that of their partners’, was confirmed. On the one hand, men’s quality-of-life was more affected by objective data such as older age, low-level education, or longstanding desire for a child. On the other hand, emotionally sensitive variables for women, such as attitudes about the cause of their failure to conceive and already having a child, related more strongly to QoL. These findings partly match the results of Huppelschoten et al., 2013 [19].

It is well-known that couples or women with secondary infertility, notably if they are already parents, often achieve higher levels of fertility-specific quality of life than those with primary fertility problems (e.g. [13, 37]. This evidence was reflected in our result indicating that women already mothering a child reported a higher level of QoL in the emotional and physical domains. However, women were more likely to report worse relational QoL if they had a child from the current relationship. Only a few studies have so far established that secondary infertility is related to poorer relational or sexual adjustment [38, 39]. It is worth remembering that couples with children have to face different challenges and more stressful situations than couples without children, a constellation that may monopolize the efforts they would otherwise expend on strengthening their relationship. It remains an interesting question for further research why it is favourable for women on personal levels to have a biological child already, but it retards experiencing QoL on the relational level.

Interestingly, poor education in men even had an impact on how women felt about fertility problems in the partnership and other social relations. Better-educated individuals have better job prospects with higher incomes, so here the links to higher quality of life levels are obvious [40]. We assume that the strong connection between education and quality of life has an effect on romantic dyads as well. Higher socioeconomic status encourages greater self-confidence and in social situations may be a kind of compensation for the frustration caused by involuntary childlessness.

On the other hand, causes of fertility impairments perceived by the female partner had cross-partner effects on men’s Emotional, Mind/Body and Relational QoLs. Both partners have been found to show less favourable psychosocial indicators when the men or women themselves were the cause of the fertility problem [41, 42]. In our study, men tend to report better Relational QoL if their wives suspect that the male factor is the sole cause of infertility. This result is unexpected and we can only suppose that it is rooted in the fact that male-factor-only infertility indicated higher QoL in women and that the male partner’s psychological adjustment is mediated via the feedback of the woman experiencing less fertility-related stress [27, 43]. Nevertheless, the combined factor perceived as the source of fertility problem was a predictor for lower Mind/Body QoL in both women and men and also for lower Emotional QoL in men. Medically, the combined factor is in itself the least promising infertility condition with the worst chances of having children of one’s own [44] and hence indicates lower levels of psychological well-being [45].

### Strengths and limitations

One of the strengths of the study is that we worked with a large sample size (*n* = 594) involving both partners of couples in 78% of the cases. Data collection was conducted at not just one, but three different fertility centres, and the response rate was high (85% for women and 76% for men).

Our analysis supported the four-factor structure for FertiQoL in a German sample. The four factors – where Emotional and Mind/Body subscales had an especially strong intercorrelation (*r* > .70) – adequately reflect the psychosocial consequences of infertility experienced by the participants at the emotional, physical, relational and social level. Our results add a new perspective to the evaluation of FertiQoL underpinning the argument that cultural aspects should be considered in the evaluation of fertility-related quality of life.

In addition, our results highlight different gender patterns discernible in the way sociodemographic factors of individuals affect QoL. In case of women, subjective attitudes (towards perceived cause of infertility, previous child) show a significant connection to fertility-related quality of life, while in men, objective factors (age, education level, duration of desire for a child) relate significantly to QoL. These relations were also strengthened at the cross-partner level, underlining the strong effect of poor education in men on women’s QoL and of women’s attitudes to the cause of infertility on men’s QoL.

The study also has a number of limitations. Generalization of the findings is restricted because of the cross-sectional design and clinical data collection. The study contains no test for the convergent validity of the instrument because only the FertiQoL questionnaire was used. The sample consisted both of independent individuals and of couples so that the differences in psychological variables between women and men should be interpreted cautiously. Further research is needed to examine the correlations of German FertiQoL with other standardized scales measuring similar psychological constructions to fertility-related quality of life [14–16].

There was no clear identification of whether the participants knew about the infertility diagnosis (no cause, female factor only, male factor only, combined factor) and had accepted it or whether their answers about the cause of fertility problems were simply based on subjective intuition. In further studies assessing personally perceived causes of infertility (not the medically confirmed diagnoses), it will be necessary to ask whether the participants had obtained information about the medical diagnosis.

## Conclusions

In conclusion, the German version of FertiQoL in both genders proved to a large extent to be a valid and reliable measure with a four-factor structure connecting up to different sociodemographic and medically relevant aspects in men and women (with the exception of an especially strong intercorrelation in Emotional and Mind/Body subscales). The use of the FertiQoL in fertility care is recommended because it can provide important information for the medical staff and the patients themselves on the challenges they face in connection with emotional, physical, relational and social quality of life. In practice, the questionnaire is a feasible instrument for appraising the way couples with fertility problems function psychosocially. It also gives counsellors the basic knowledge they require to provide individually tailored psychosocial care for the couples and (ideally) to help them achieve their parental goals. At the same time, further research is needed to verify the cross-country and cross-cultural validity of the instrument in order to improve FertiQoL as an internationally valid tool [24].

## Abbreviations

ART: Assisted Reproductive Technologies
AVE: Average Variances extracted
CFA: Confirmatory Factor Analysis
CFI: Comparative Fit Index
QoL: Quality of Life
RMSEA: Root-Mean-Square Error of Approximation
SRMR: Standardized Root-Mean-Square Residuals
WHO-HRQOL: World Health Organisation-Health related Quality of Life.

## References

[1] Inhorn MC, Patrizio P. Infertility around the globe: new thinking on gender, reproductive technologies and global movements in the 21st century. Hum Reprod Update. 2015. 10.1093/humupd/dmv016 25801630

[2] Mascarenhas MN, Flaxman SR, Boerma T, Vanderpoel S, Stevens GA (2012). National, regional, and global trends in infertility prevalence since 1990: a systematic analysis of 277 health surveys. PLoS Med.

[3] Boivin J, Bunting L, Collins JA, Nygren KG (2007). International estimates of infertility prevalence and treatment-seeking: potential need and demand for infertility medical care. Hum Reprod.

[4] De Geyter C, Calhaz-Jorge C, Kupka MS, Wyns C, Mocanu E, Motrenko T, Scaravelli G, Smeenk J, Vidakovic S, Goossens V, et al: ART in Europe, 2014: results generated from European registries by ESHRE†The European IVF-monitoring Consortium (EIM)‡ for the European Society of Human Reproduction and Embryology (ESHRE). Hum Reprod 2018:dey242-dey242. 10.1093/hropen/hoaa038 PMC7508022 32995563

[5] Gameiro S, Boivin J: The Psychology of Infertility in Reproductive Medicine and Healthcare, c. 1940s–2000s. In The Palgrave Handbook of Infertility in History: Approaches, Contexts and Perspectives*.* Edited by Davis G, Loughran T. London: Palgrave Macmillan UK; 2017: 393–413.

[6] Greil AL (1997). Infertility and psychological distress: a critical review of the literature. Soc Sci Med.

[7] Pasch LA, Sullivan KT (2017). Stress and coping in couples facing infertility. Curr Opin Psychol.

[8] Verhaak CM, Smeenk JMJ, Evers AWM, Kremer JAM, Kraaimaat FW, Braat DDM (2007). Women’s emotional adjustment to IVF: a systematic review of 25 years of research. Hum Reprod Update.

[9] Gameiro S, Boivin J, Peronace L, Verhaak CM (2012). Why do patients discontinue fertility treatment? A systematic review of reasons and predictors of discontinuation in fertility treatment. Hum Reprod Update.

[10] Greil AL, Slauson-Blevins K, McQuillan J (2010). The experience of infertility: a review of recent literature. Sociology of Health & Illness.

[11] Gameiro S, Boivin J, Dancet E, de Klerk C, Emery M, Lewis-Jones C, Thorn P, Van den Broeck U, Venetis C, Verhaak CM (2015). ESHRE guideline: routine psychosocial care in infertility and medically assisted reproduction—a guide for fertility staff. Hum Reprod.

[12] Boivin J: Psychological Fertility-Related Questionnaires. In Infertility counseling A comprehensive handbook for clinicians*.* 2 edition. Edited by Covington SN, Burns LH. Oxford London New York: Taylor & Francis; 2006: 565–568.

[13] Boivin J, Takefman J, Braverman A (2011). The fertility quality of life (FertiQoL) tool: development and general psychometric properties. Fertil Steril.

[14] Melo C, Gameiro S, Canavarro MC, Boivin J (2012). Does the FertiQoL Assess Quality of Life? Results from the Validation of the Portuguese Version of the FertiQoL. Hum Reprod.

[15] Donarelli Z, Lo Coco G, Gullo S, Salerno L, Marino A, Sammartano F, Allegra A. The fertility quality of life questionnaire (FertiQoL) relational subscale: psychometric properties and discriminant validity across gender. Hum Reprod. 2016;31:2061-2071. 10.1093/humrep/dew168 27343271

[16] Aarts JWM, van Empel IWH, Boivin J, Nelen WL, Kremer JAM, Verhaak CM (2011). Relationship between quality of life and distress in infertility: a validation study of the Dutch FertiQoL. Hum Reprod.

[17] Dural O, Yasa C, Keyif B, Celiksoy H, Demiral I, Yuksel Ozgor B, Gungor Ugurlucan F, Bastu E (2016). Effect of infertility on quality of life of women: a validation study of the Turkish FertiQoL. Hum Fertil.

[18] Maroufizadeh S, Ghaheri A, Amini P, Samani RO (2017). Psychometric properties of the fertility quality of life instrument in infertile Iranian women. Int J Fertil Steril.

[19] Huppelschoten AG, van Dongen AJCM, Verhaak CM, Smeenk JMJ, Kremer JAM, Nelen WLDM (2013). Differences in quality of life and emotional status between infertile women and their partners. Hum Reprod.

[20] Hsu P-Y, Lin M-W, Hwang J-L, Lee M-S, Wu M-H (2013). The fertility quality of life (FertiQoL) questionnaire in Taiwanese infertile couples. Taiwanese J Obstet Gynecol.

[21] Karabulut A, Özkan S, Oğuz N (2013). Predictors of fertility quality of life (FertiQoL) in infertile women: analysis of confounding factors. European J Obstet Gynecol Reprod Biol.

[22] Keramat A, Masoomi SZ, Mousavi SA, Poorolajal J, Shobeiri F, Hazavhei SM (2014). Quality of life and its related factors in infertile couples. J Res Health Sci.

[23] Porat-Katz A, Paltiel O, Kahane A, Eldar-Geva T (2016). The effect of using complementary medicine on the infertility-specific quality of life of women undergoing in vitro fertilization. Int J Gynecol Obstet.

[24] Sexty R, Hamadneh J, Rosner S, Strowitzki T, Ditzen B, Toth B, Wischmann T (2016). Cross-cultural comparison of fertility specific quality of life in German, Hungarian and Jordanian couples attending a fertility center. Health Qual Life Outcomes.

[25] Chachamovich J, Chachamovich E, Fleck MP, Cordova FP, Knauth D, Passos E (2009). Congruence of quality of life among infertile men and women: findings from a couple-based study. Hum Reprod.

[26] Donarelli Z, Lo Coco G, Gullo S, Marino A, Volpes A, Allegra A (2012). Are attachment dimensions associated with infertility-related stress in couples undergoing their first IVF treatment? A study on the individual and cross-partner effect. Hum Reprod.

[27] Martins MV, Peterson BD, Almeida V, Mesquita-Guimarães J, Costa ME (2014). Dyadic dynamics of perceived social support in couples facing infertility. Hum Reprod.

[28] Volmer L, Rösner S, Toth B, Strowitzki T, Wischmann T (2017). Infertile Partnersʼ coping strategies are interrelated – implications for targeted psychological counseling. Geburtshilfe Frauenheilkd.

[29] L-t H, Bentler PM (1999). Cutoff criteria for fit indexes in covariance structure analysis: conventional criteria versus new alternatives.

[30] Fornell C, Larcker DF (1981). Evaluating structural equation models with unobservable variables and measurement error. J Mark Res.

[31] Tavakol M, Dennick R (2011). Making sense of Cronbach’s alpha. Int J Med Educ.

[32] Lund R, Sejbaek CS, Christensen U, Schmidt L. The impact of social relations on the incidence of severe depressive symptoms among infertile women and men. Hum Reprod. 2009;24:2810-2820.. 10.1093/humrep/dep257 19625314

[33] Hjelmstedt A, Andersson L, Skoog-SVANBERG A, Bergh T, Boivin J, Collins A (1999). Gender differences in psychological reactions to infertility among couples seeking IVF- and ICSI-treatment. Acta Obstet Gynecol Scand.

[34] Cserepes RE, Bugan A, Korosi T, Toth B, Rosner S, Strowitzki T, Wischmann T (2014). Infertility specific quality of life and gender role attitudes in German and Hungarian involuntary childless couples. Geburtshilfe Frauenheilkd.

[35] Madero S, Gameiro S, Garcia D, Cirera D, Vassena R, Rodriguez A (2017). Quality of life, anxiety and depression of German, Italian and French couples undergoing cross-border oocyte donation in Spain. Hum Reprod.

[36] Skevington SM, Lotfy M, O'Connell KA (2004). The World Health Organization’s WHOQOL-BREF quality of life assessment: psychometric properties and results of the international field trial. A report from the WHOQOL group. Qual Life Res.

[37] Newton CR, Sherrard W, Glavac I (1999). The fertility problem inventory: measuring perceived infertility-related stress. Fertil Steril.

[38] Keskin U, Coksuer H, Gungor S, Ercan CM, Karasahin KE, Baser I (2011). Differences in prevalence of sexual dysfunction between primary and secondary infertile women. Fertil Steril.

[39] Pakpour AH, Yekaninejad MS, Zeidi IM, Burri A (2012). Prevalence and risk factors of the female sexual dysfunction in a sample of infertile Iranian women. Arch Gynecol Obstet.

[40] Quality of Life Indicators - Education [https://ec.europa.eu/eurostat/statistics-explained/index.php?title=Quality_of_life_indicators_-_education].

[41] Lee TY, Sun GH, Chao SC (2001). The effect of an infertility diagnosis on the distress, martial and sexual satisfaction between husband and wives in Taiwan. Hum Reprod.

[42] Nachtigall RD, Becker G, Wozny M (1992). The effects of gender-specific diagnosis on men's and women's response to infertility. Fertil Steril.

[43] Greil AL, Slauson-Blevins K, McQuillan J, Lowry MH, Burch AR, Shreffler KM (2018). Relationship satisfaction among infertile couples: implications of gender and self-identification. J Fam Issues.

[44] Evers J (2002). Female subfertility. Lancet.

[45] Heredia M, Tenías JM, Rocio R, Amparo F, Calleja MA, Valenzuela JC (2013). Quality of life and predictive factors in patients undergoing assisted reproduction techniques. European J Obstet Gynecol Reprod Biol.

